# Relevance of hemostatic risk factors on coronary morphology in patients with diabetes mellitus type 2

**DOI:** 10.1186/1475-2840-8-24

**Published:** 2009-05-06

**Authors:** Thomas W Jax, Ansgar J Peters, Gunnar Plehn, Frank-Chris Schoebel

**Affiliations:** 1 Profil Institut für Stoffwechselforschung, Division of Cardiometabolic Research, Hellersbergstrasse 9, 41460 Neuss, Germany; 2 Klinik für Kardiologie, Herzzentrum Wuppertal, Universität Witten-Herdecke, Wuppertal, Germany; 3 Medizinische Klinik und Poliklinik B (Internal Medicine), Department of Cardiology, Pneumology and Angiology, Heinrich-Heine-University, Düsseldorf, Germany; 4 Klinik für Kardiologie, Marienhospital Herne, Herne, Germany

## Abstract

**Objective:**

The influence hemostatitc parameters on the morphological extent and severity of coronary artery disease were studied in patients with and without DM type 2.

**Background:**

It is known that patients with diabetes (DM) have abnormal metabolic and hemostatic parameters

**Methods:**

Of 150 consecutive patients with angiographically proven coronary artery disease 29 presented with DM. Additionally to parameters of lipid-metabolism fibrinogen, tissue-plasminogenactivator (t-PA), plasminogen-activator-inhibitor (PAI), plasmin-a-antiplasmin (PAP), prothrombin-fragment 1+2 (F1+2), thrombin-antithrombin (TAT), von-willebrand-factor (vWF), platelet factor 4 (PF4), glykomembranproteine 140 (GMP140) and the rheologic parameters plasma viscosity and red blood cell aggregation were evaluated. The extent and severity of CAD was evaluated according to the criteria of the American Heart Association.

**Results:**

Patients with DM presented with a higher number of conventional risk factors as compared to non-diabetic patients. Additionally there were significant differences for F1+2, red blood cell aggregation and PAI. Diabetic patients showed a more severe extent of coronary arteriosclerosis, which also could be found more distally. A significant relationship between blood-glucose, thrombocyte-activation (vWF), endogenous fibrinolysis (PAI) and the severity of CAD and a more distal location of stenoses could be found (r = 0.6, p < 0.001).

**Conclusion:**

Patients with coronary artery disease and DM type 2 showed marked alterations of metabolic, hemostatic, fibrinolytic and rheologic parameters, which can produce a prothrombogenic state. A direct association of thrombogenic factors on coronary morphology could be shown. This can be the pathophysiologic mechanism of more severe and distal pronounced coronary atherosclerosis in these patients.

## Introduction

Coronary artery disease is the essential cardiac manifestation of diabetes mellitus type 2 and is responsible for the predominant number of deaths. Early autopsy studies pointed out, that the morphological manifestation of coronary artery disease in patients with diabetes mellitus increases three to four times as compared with non-diabetics [1-3]. The clinical relevance of these results is documented by an increased incidence of cardiovascular events, and particularly in the "Framingham study" [4] and in the following epidemiological and longitudinal examinations [5,6]. The influence of diabetes mellitus is statistically independent of other established risk factors for the manifestation of coronary heart disease and is regarded as the risk factor with the highest predictive value for the incidence of cardiovascular complications

Since coronary thrombosis is essential for the pathogenesis of most acute coronary syndromes [7], the prothrombogenic constellation is probably of importance for the increased cardiovascular event rate among diabetics. Furthermore it seems likely, that subclinical intracoronary thrombi may promote atherosclerotic changes in patients with coronary heart disease [8].

During the last few years increased attention has been given to the complex metabolic changes associated with diabetes mellitus type 2 and insulin resistance. In this study, metabolic changes associated with diabetes, particularly changes of hemostasis and endogenous fibrinolysis were studied. The importance with regard to the incidence of cardiovascular events for diabetic patients was already proven in large epidemiological studies [9-14]; the direct association with morphological changes was not assessed yet.

In the present study we test the hypothesis that a) diabetic patients show a more diffuse form of coronary artery disease with a higher rate of distal stenosis and that b) haemostatic parameters correlate with the extent of coronary atherosclerosis in patients with diabetes mellitus type 2

## Patients, material and methods

For this study 150 consecutive patients aged between 50 and 65 years, who were scheduled for elective coronary angiography, were recruited at Heinrich-Heine-University, Division of Cardiology. Unstable angina pectoris, acute myocardial infarction within the last six months before inclusion into the study, and prior coronary interventions were exclusion criteria. Furthermore, patients with acute or chronic infections and malignant diseases were excluded. Patients with a diabetes type 1 were not included in the study.

### Definition of cardiovascular risk factors

Hyperlipidemia was defined as with total cholesterol above 220 mg/dl, as triglycerides above 200 mg/dl, with a positive history of hypercholesterolemia or hypertriglyceridemia and with lipid reducing therapies.

Diabetes was defined as fasting blood glucose above 126 mg/dl, or antidiabetic therapy. Hypertension was defined as resting blood pressure above 140 mmHg systolic or 90 mmHg diastolic, or with antihypertensive medication. Nicotine consumption was assessed by patient history.

### Coronary Angiography

In all patients coronary angiograms with imaging of the coronary arteries in different standardised projections were carried out. The degree of coronary artery stenosis was assessed by two experienced, independent examiners. The severity of CAD was defined as coronary 1, 2 or 3 vessel disease corresponding to the number of affected main vessels with hemodynamic relevant stenosis above 50%. Extent of coronary atherosclerosis was determined by a special grading system in accordance with an evaluation system of the American Heart Association [15]. Based on these findings the coronary artery system was divided into 15 segments. The segment with a higher degree of stenosis got a higher score. The sum of these scores was divided by the number of judged segments. The result presents the extent of coronary atherosclerosis. To get information about the distribution pattern of coronary atherosclerosis a proximal score from the proximal segments of coronary main arteries and a distal score from the distal parts of main coronary arteries and vessels of first degree were calculated.

### Laborchemical methods

Blood samples for the different parameters were taken at the day of cardiac catheterisation between 7:00 and 8:00 am after 30 minutes rest in a lying position. Venous puncture of an antecubital vene was done with a butterfly canula after a maximum venous compression time of 30 seconds and a maximum pressure of 40 mmHg. After decompression the first 4 ml of blood were not used. For parameters of hemostasis and endogenous fibrinolysis vacutainer tubes with 0.129 M Na_2 _citrat were used. Tubes underwent centrifugation at 2000 g for 15 minutes. The plasma was taken and centrifugated at 2000 g for another 15 minutes. Samples were frozen at -80°C. The samples were analysed immediately after preparation from deeply frozen plasma within three months after sample taking.

Determination of plasma viscosity and red blood cell aggregation was performed from EDTA tubes within 4 hours after sample taking. Plasmaviscosity was determined by the capillary tube viscosimeter of Rheomed, Aachen (Germany). For determination of red blood cell aggregation an erythrocyte aggregometer (Myrenne, Roetgen, Germany) was used.

Plasma fibrinogen levels were determined according to method described by Clauss [16]. Prothrombin fragment 1 and 2 were measured by Elisa. Specific activity of AT III was measured by a cinetic test; Protein C and S were measured by coagulometric tests. Determination of Plasminogenactivator inhibitor activity was performed by chromogenic enzymassay. Thrombin-Antithrombin III complex, plasma tissue plasminogenactivator antigen concentration, plasmin a2 antiplasmin and d-dimeres were determined by enzymimmunoassay.

### Obesity indices

As a calculated index to estimate the extent of Obesity the "body mass index" is clinically established. This index calculates itself by the body weight (kg) being divided by the square of the height (m^2^). The relationship between waists and hip circumference forms another parameter for the classification of obesity [17]. This quotient was shown to correlate with the visceral fat mass [18].

### Statistical methods

Statistical analyses were performed using SPSS software (Statistical Package for Social Sciences for Windows; SPSS 12.0, Munich). The results were tested for normal distribution by Kolmogoroff-Smirnoff test. For normally distributed parameters group comparison was made by use of paired and unpaired Student's t-test. Mann-Whitney U-Test was used for non-normally distributed parameters. The Pearson correlation coefficient or the Spearman's rank correlation was used to describe the association between the variables in the study. A stepwise regression model was used to test the relationship of laboratory parameters on morphology. Chi square test was used for comparison of non-continuous data. Statistical significance was defined as p < 0.05.

## Results

Out of 150 consecutive patients, 28 patients presented with the diagnosis of diabetes mellitus type 2 (19%). Both groups showed a comparable age profile, they were not different with regard to height, however, diabetic patients were heavier, what resulted in a significantly higher "body mass index". Both waist circumference and the "Waist-to hip ratio" calculated from this differed significantly.

Diabetic patients showed a 20% higher rate of arterial hypertension (table 1). Other conventional risk factors did not differ between the groups (table 1). The laboratory evaluation showed that only HDL cholesterol differed significantly, although the differences for triglycerides are only not significantly different because of the wide standard deviation (table 1). Parameters of glucose metabolism were markedly elevated in diabetic patients, as expected; there was no difference in fasting insulin levels.

**Table 1:** Patient characteristics

	all patients (n = 150)	Diabetes mellitus Typ II (n = 26)	control patients (n = 122)	p-value
age (years)	58 ± 5	57 ± 4	58 ± 5	0.1158
hypertension (%)	62.0%	85.00%	57.00%	0.0076
hypercholesterinemia (%)	74.0%	73.00%	74.00%	0.9419
hypertriglyceridemia(%)	40.0%	46.00%	39.00%	0.47068
smoking (%)	10.0%	4.00%	11.00%	0.28139
ex-smoking (%)	75.0%	81.00%	74.00%	0.4543
history of vascular diesease (%)	31.0%	39%	30.00%	0.37048
obesity (%)	83.0%	100%	80.00%	0.01134
Hyperurikämie (%)	20.0%	12%	22.00%	0.26987
				
total cholesterol (mg/dl)	226 ± 42	225 ± 50	225 ± 40	0.5254
HDL – cholesterol (mg/dl)	44 ± 13	37 ± 10	46 ± 14	0.0035
LDL – cholesterol (mg/dl)	157 ± 39	159 ± 45	156 ± 37	0.9246
triglycerides (mg/dl)	210 ± 141	239 ± 189	205 ± 130	0.4681
lipoprotein (a) (mg/dl)	20,4 ± 22,5	16,1 ± 21,9	20,7 ± 22,1	0.1302
uric acid (mg/dl)	6,3 ± 1,5	6,4 ± 1,6	5,6 ± 1,1	0.0341
fasting plasma glucose (mg/dl)	116 ± 51	197 ± 75	100 ± 19	0.0001
Insuline (mU/l)	8,8 ± 5,7	8,7 ± 4,7	8,8 ± 5,9	0.7382
HbA1c (%)	6,0 ± 1,3	8,1 ± 1,5	5,5 ± 0,6	0.0001
				
heigth	174 ± 6	173 ± 7	174 ± 6	0.4923
weight (kg)	83 ± 12	85 ± 10	82 ± 12	0.171
Body-Mass-Index (kg/m2)	27,35 ± 3,42	28,42 ± 2,78	27,12 ± 3,51	0.0367
Waist-to-Hip-Ratio	0,99 ± 0,07	1,03 ± 0,07	0,98 ± 0,07	0.0035
waist circumference (cm)	99 ± 9	104 ± 9	100 ± 6	0.0079
hip circumference (cm)	100 ± 6	100 ± 7	100 ± 6	0.6175

Patients with diabetes mellitus type 2 showed higher values for fibrinogen, the difference, however, was not significant (table 2). Also, prothrombin fragments 1 and 2 as indicators for a thrombin synthesis and a marker for prothrombotic conditions, was elevated in patients with diabetes mellitus, although again, not significantly. Anthrombin III and D-dimer showed no statistical significant differences (table 2). Plasma viscosity and aggregation of erythrocytes as the essential variables influencing microcirculation were higher in patients with diabetes mellitus. Of the markers of fibrinolysis PAI activity was significantly higher in patients with diabetes. Thrombocytic parameters did not differ at all between the two groups.

**Table 2:** Parameters of hemostasis, rheology, fibrinolysis, platelet activation and extent and severity of coronary atherosclerosis

	all patients	Diabetes mellitus Typ II	control patients	p-value
	(n = 150)	(n = 26)	(n = 122)	
				
*hemostasis*				
fibrinogen (mg/dl)	282 ± 64	295 ± 76	278 ± 61	0.1618
prothrombinfragment 1 + 2 (μg/dl)	0,99 ± 0,52	1,01 ± 0,55	0,87 ± 0,37	0.0622
D-Dimere (μg/l)	35 ± 62	31 ± 43	36 ± 65	0.5519
Thrombin-Antithrombin-Komplex (μg/l)	7,6 ± 16	9,6 ± 18,7	7,3 ± 15,5	0.5221
				
*Rheology*				
plasma viscosity (mPas)	1,30 ± 0,09	1,33 ± 0,09	1,29 ± 0,09	0.0933
erythocyte aggregation M (E)	6,3 ± 1,8	7,3 ± 1,9	6,1 ± 1,8	0.0026
erythocyte aggregation M1 (E)	11,3 ± 2,5	12,8 ± 2,7	11,0 ± 2,3	0.0013
hematocrit (%)	43,5 ± 3,1	42,7 ± 3,7	43,6 ± 2,9	0.1537
				
*fibrinolysis*				
tissue-plasminogenaktivator (ng/dl)	13,47 ± 3,68	14,18 ± 3,36	13,30 ± 3,77	0.4394
plasminogenactivatorinhibitor (U/ml)	5,78 ± 3,25	7,36 ± 3,24	5,45 ± 3,18	0.0017
plasmin-α2-antiplasmin-complex (μg/dl)	395 ± 152	378 ± 168	396 ± 146	0.4245
				
*platelet activation*				
platelet count (100.000/ml)	246 ± 71	265 ± 71	242 ± 71	0.1727
von Willebrand-factor (μg/l)	94 ± 31	99 ± 32	93 ± 30	0.3661
plateletfactor 4 (μg/l)	12,7 ± 18,8	9,2 ± 10,9	13,4 ± 20,1	0.9104
β-thromboglobulin (μg/l)	36,68 ± 41,90	30,07 ± 17,57	38,28 ± 45,53	0.7181
GMP 140 (μg/l)	96,36 ± 44,69	94,64 ± 23,41	96,88 ± 48,26	0.2773
				
*Extent and severity of coronary atherosclerosis*				
total score	1,451 ± 0,591	1,858 ± 0,680	1,379 ± 0,550	0.0182
proximal score	1,399 ± 0,696	1,775 ± 1,037	1,333 ± 0,607	0.2363
distal score	1,457 ± 0,666	1,874 ± 0,647	1,388 ± 0,651	0.0109
				
1-vessel CAD	31.0%	12.0%	35.0%	
2-vessel CAD	37.0%	39.0%	37.0%	
3-vessel CAD	32.0%	50.0%	28.0%	0.0306

Patients with diabetes more often presented with 3 vessel disease (table 2). The evaluated coronary score was significantly higher in patients with than without diabetes mellitus. Patients with diabetes especially showed a higher distal score.

The plasma levels of insulin and HbA1c correlated on the one hand significantly with PAI activity and tPA concentration, on the other hand also with indices of obesity (table 3). Of the parameters of lipid metabolism only triglycerides correlated with insulin. Fasting glucose on the day of coronary angiography strongly correlated with the total and the distally weighted score (R = 0.48, table 3).

**Table 3:** The Pearson correlation coefficient or the Spearman's rank correlations were used to describe the association between the variables in the study.

	tPA	PAI	cholesterol	HDL	LDL	tg	fibrinogen	plasmaviscosity
**insuline**	0.3173	0.2573	0.0112	-0.152	0.0304	0.2702	-0.0011	0.0444
	0.008	0.002	0.896	0.076	0.725	0.001	0.99	0.612
**HbA1c**	0.2941	0.4117						
	0.016	0.0001						
								
	**insuline**	**HbA1c**	**HDL**	**LDL**				
**WHR**	0.2716	0.2903	-0.3765	0.3134				
	0.033	0.014	0.002	0.009				
**C-Index**	0.4067	0.4429	-0.3539	0.1792				
	0.001	0.0001	0.004	0.153				
								
	**total score**	**prox. score**	**dist. score**					
**glucose**	0.4872	0.2097	0.4769					
	0.0001	0.108	0.4769					

Upper line denotes R, lower line the according p-value

We then calculated a univariant regression model for each of the three coronary scores and parameters of lipid and glucose metabolism, hemostasis and fibrinolysis, platelet activation, rheology, obesity and hypertension. Results are given in table 4. All significant parameters were included into a multivariate, stepwise regression analysis. This analysis revealed the most significant correlation between fasting glucose and all coronary scores but in particular with total and distal score, followed by waist to hip ratio and PAI activity for the distal score and von-willebrand factor for total score (table 4)

**Table 4:** univariant and multivariant (stepwise backward) regression model for each of the three coronary scores and parameters of lipid and glucose metabolism, hemostasis and fibrinolysis, platelet activation, rheology, obesity and hypertension.

	total score			distal score			proximal score	
	Univariat		Multivariat	Univariat		Multivariat	Univariant	
	R	p-value		R	p-value	p-value	R	p-value
*Conventional risk factors*								
Total cholesterol (mg/dl)	0,17099	0,1665		0,15137	0,2214		0,12198	0,3254
HDL – cholesterol (mg/dl)	0,1864	0,134		0,21858	**0,0779**	0,3475	0,02781	0,8246
LDL – cholesterol (mg/dl)	0,11409	0,3617		0,08219	0,5118		0,11332	0,365
Triglycerides (mg/dl)	0,00269	0,9827		0,00918	0,9412		0,02211	0,8591
Uric acid (mg/dl)	0,28913	**0,0205**	0,2187	0,28087	**0,0246**	0,1682	0,16794	0,2011
Plasma glucose (mg/dl)	0,5456	**0,0001**	**0,0001**	0,47045	**0,0001**	**0,0001**	0,42108	**0,0005**
Insuline (mU/l)	0,01736	0,8943		0,01917	0,8834		0,10553	0,4182
HbA1c (%)	0,13988	0,2589		0,14837	0,2308		0,02186	0,8606
								
*Hemostasis*								
Fibrinogen (mg/dl)	0,07329	0,5556		0,07759	0,5326		0,03252	0,7939
Prothrombinfragment 1+ 2 (μg/l)	0,05127	0,6803		0,05278	0,6715		0,00098	0,9937
								
D-dimers (μg/l)	0,22375	0,0687		0,19556	0,1127		0,19572	0,1124
Thrombin-Antithrombin-complex	0,11095	0,3714		0,10142	0,4141		0,07338	0,5551
tissue-Plasminogenactivator (ng/dl)	0,09567	0,4913		0,07875	0,5714		0,08	0,5653
Plasminogenactivatorinhibitor (U/ml)	0,22549	**0,0666**	0,1947	0,25669	**0,036**	**0,0407**	0,03477	0,78
Plasmin-a2-Antiplasmin-complex (μg/dl)	0,08721	0,4828		0,08614	0,4882		0,05627	0,6511
								
*Platelet parameters*								
von Willebrand-Factor (μg/l)	0,3335	**0,0058**	**0,013**	0,32558	**0,0072**	0,2661	0,2229	0,698
Platelet factor 4 (μg/l)	0,00077	0,9951		0,02395	0,8486		0,6825	0,5861
β-Thromboglobulin (μg/l)	0,08704	0,4871		0,05816	0,6428		0,11954	0,339
GMP 140 (μg/l)	0,11087	0,3718		0,10535	0,3962		0,07522	0,5452
								
*Rheological parameters*								
Plasma viscosity (mPas)	0,05615	0,6518		0,07809	0,5299		0,0132	0,9156
Erythrocyte aggregation M (E)	0,00747	0,9552		0,02073	0,8761		0,02058	0,8771
Erythrocyte aggregation M1 (E)	0,02564	0,8471		0,04427	0,7392		0,034	0,7982
								
*Obesitys-Indices*								
BMI (kg/m^2^)	0,07228	0,561		0,06516	0,6003		0,06279	0,6137
WHR	0,18316	0,165		0,24149	**0,0654**	**0,0003**	0,00075	0,9955
Waist (cm)	0,02086	0,8799		0,01037	0,9401		0,05692	0,6798
Hip(cm)	0,00135	0,9922		0,00294	0,983		0,02922	0,8323
								
*Blood pressure*								
systolic (mm Hg)	0,14903	0,2287		0,14472	0,2426		0,10775	0,3855
diastolic (mm Hg)	0,10406	0,402		0,05629	0,6509		0,16688	0,1771
mean (mm Hg)	0,1775	0,1507		0,13861	0,2633		0,19968	0,1052

## Discussion

### Coronary morphology in diabetes mellitus

Although it is well established, that type 2 diabetes is a strong and independent risk factor for the development of atherosclerosis, there is much discussion about a "typical", diabetes related morphology of atherosclerosis. Many clinicians today believe, that diabetic patients present with more extensive atherosclerotic disease than non-diabetics. However, to our knowledge only very few studies confirm these subjective impressions, and these are mostly autopsy based. Early autopsy studies pointed out, that the morphological manifestation of coronary artery disease in patients with diabetes mellitus increases three to four times as compared with non-diabetics [1-3].

Here, we present a systematic approach to evaluate the coronary morphology of consecutive patients by angiography with regard to their diabetic status and can confirm this widespread belief. Coronary atherosclerosis can be found in more segments and is generally located more distally. This also confirms the results of a recent study, which could show a higher extent of coronary plaque burden in diabetic patients [19]. Recent studies using intravascular ultrasound (IVUS) to evaluate coronary plaque burden in diabetic patients could show more extensive atherosclerosis [20-22]. However, these studies are often technically limited and can not be used to assess distal segments. Nicholls and co-workers assessed IVUS and also quantitative coronary angiography (QCA) and could not find a correlation between diabetes and stenosis severity. However, QCA was only done in segments also evaluated by intravascular ultrasound and did only comprise the most severe stenosis per target vessel [22]. They did find diabetes to be a strong independent predictor of atherosclerotic coronary disease, but the distribution of atherosclerosis within the coronary arteries was not assessed. An analysis of pooled data revealed that coronary artery atherosclerosis in patients with diabetes mellitus is more extensive and accompanied by inadequate compensatory remodelling [23]. Additionally the progression of atheroma was more rapid in patients with diabetes, despite high use of well established and potent therapies. With this in mind angiographic assessment could underestimate the plaque burden. On the other hand recent studies suggest, that a high percentage of cardiac patients present with newly diagnosed type 2 diabetes by use of oral glucose tolerance tests (OGTT) [24-26]. OGTT was not part of this protocol; therefore the true rate of diabetes may be underestimated.

### Hemostasis and fibrinolysis in diabetes mellitus

Type 2 diabetes mellitus, most often being part of a metabolic syndrome is closely associated with obesity, peripheral insulin resistance, arterial hypertension, hypercholesterinemia, hypertriglyceridemia and predisposes for atherosclerosis [27]. Peripheral insulin resistance is strongly associated with atherosclerosis, even after adjusting for other more conventional risk factors [28]. Whether this relation is causal, can only be suspected [28,29], other authors discussed whether an impaired fibrinolysis could be the link to atherosclerosis [30].

The changes of hemostasis and endogenous fibrinolysis of diabetic patients in this study correspond with the literature [9-13,30], although most often only single parameters were evaluated. These alterations might be responsible for higher atherogenicity and more cardiovascular events in a diabetic population.

We showed, that plasminogen activator inhibitor activity (PAI) was increased in diabetic patients; this corresponds to other studies [12,14,31,32]. Additionally we found a strong correlation between PAI and insulin levels, which confirms results from experimental studies, where insulin decreased PAI activity in a dose dependent manner [12,33].

The potential role of insulin as one of the mechanisms in atherogenesis in diabetic patients has been discussed for 25 years [34]. Results of various prospective studies indicated that elevated plasma insulin levels cause and enhance atherosclerosis [35-37]. Diverse diseases associated with hyperinsulinemia show an early and extended manifestation of atherosclerosis [38-43]. Several studies proved that elevated insulin levels are accompanied by reduced endogenous fibrinolysis [12,14,31]. On the one hand hyperinsulinemia is associated with a decreased t-PA concentration [11], on the other hand it causes an increase of PAI activity [12-14,31].

The importance of PAI is underlined by a follow up study of men after primary myocardial infarction. Those patients who showed elevated PAI concentrations as a sign of reduced fibrinolysis were at increased risk of reinfarction [44,45]. High concentrations of von Willebrand factor antigen are supposed to be markers of endothelial lesions and present a procoagulative mechanism due to an enhanced platelet adhesion [46].

The question remains, how does diabetes mediate its specific effects towards a diabetic specific morphology? Some authors suggest that diabetics undergo invasive evaluation later than others due to their cardiac neuropathy and the resulting lack of symptoms, others, that the impact of the often combined risk factors (e.g. hypertension, high LDL etc.) may trigger an accelerated form of atherosclerosis. This study extends these views by showing a close association between coronary morphology and a diabetic status with alterations of haemostasis and fibrinolysis, especially for distal coronary artery disease. Univariate and multivariate regression analysis revealed a strong relationship between the extent of coronary and especially distal coronary atherosclerosis and fasting glucose, PAI-1 and von-Willebrand-factor.

## Conclusion

Both parameters of endogenous fibrinolysis and markers of platelet activity showed a relevant association on the extent of coronary atherosclerosis. This association applied to both the total and in particular to the distally weighted coronary atherosclerosis score. Additional predisposing factors were increased uric acid and glucose concentrations as well as the "Waist hip ratio". These changes are part of the "metabolic syndrome" describing obesity, arterial hypertension and typical changes of glucose and lipid metabolism, hemostasis and fibrinolysis.

The metabolic changes with imbalance of platelet activity, hemostasis and endogenous fibrinolysis, and rheology favour a prothrombotic milieu. These systemic changes may result in an increased, thrombosis driven atherosclerotic process, in part explaining the advanced coronary plaque burden and in particular the more distal pronounced distribution of coronary atherosclerosis in patients with diabetes. More research is needed to elucidate these potential mechanisms and possibly develop a tailored therapy for these diabetes specific mechanisms of atherothrombotic disease.

## Competing interests

The authors declare that they have no competing interests.

## Authors' contributions

TJ participated in the design and coordinated the study, performed the statistical analysis and drafted the manuscript. AP coordinated the study, and drafted the manuscript. GP coordinated the study and drafted the manuscript. FS participated in the design and coordinated the study, and drafted the manuscript. All authors read and approved the final manuscript.
